# Protein-truncating variants in *BSN* are associated with severe adult-onset obesity, type 2 diabetes and fatty liver disease

**DOI:** 10.1038/s41588-024-01694-x

**Published:** 2024-04-04

**Authors:** Yajie Zhao, Maria Chukanova, Katherine A. Kentistou, Zammy Fairhurst-Hunter, Anna Maria Siegert, Raina Y. Jia, Georgina K. C. Dowsett, Eugene J. Gardner, Katherine Lawler, Felix R. Day, Lena R. Kaisinger, Yi-Chun Loraine Tung, Brian Yee Hong Lam, Hsiao-Jou Cortina Chen, Quanli Wang, Jaime Berumen-Campos, Pablo Kuri-Morales, Roberto Tapia-Conyer, Jesus Alegre-Diaz, Inês Barroso, Jonathan Emberson, Jason M. Torres, Rory Collins, Danish Saleheen, Katherine R. Smith, Dirk S. Paul, Florian Merkle, I. Sadaf Farooqi, Nick J. Wareham, Slavé Petrovski, Stephen O’Rahilly, Ken K. Ong, Giles S. H. Yeo, John R. B. Perry

**Affiliations:** 1 MRC Epidemiology Unit and NIHR Cambridge Biomedical Research Centre, Wellcome-MRC Institute of Metabolic Science, University of Cambridge School of Clinical Medicine, Cambridge, UK; 2 Metabolic Research Laboratories, MRC Metabolic Diseases Unit and NIHR Cambridge Biomedical Research Centre, Institute of Metabolic Science, University of Cambridge School of Clinical Medicine, Cambridge, UK; 3 Centre for Genomics Research, Discovery Sciences, BioPharmaceuticals R&D, AstraZeneca, Cambridge, UK; 4 Experimental Medicine Research Unit, Faculty of Medicine, National Autonomous University of Mexico, Copilco Universidad, Mexico City, Mexico; 5 Instituto Tecnológico de Estudios Superiores de Monterrey, Tecnológico, Monterrey, Mexico; 6 Exeter Centre of Excellence for Diabetes Research (EXCEED), University of Exeter Medical School, Exeter, UK; 7 MRC Population Health Research Unit, Nuffield Department of Population Health, University of Oxford, Oxford, UK; 8 Clinical Trial Service Unit & Epidemiological Studies Unit, Nuffield Department of Population Health, University of Oxford, Oxford, UK; 9 Center for Non-Communicable Diseases, Karachi, Pakistan; 10 Department of Medicine, Columbia University Irving Medical Center, New York, NY USA; 11 Institute of Metabolic Science and Cambridge Stem Cell Institute, University of Cambridge, Cambridge, UK

**Keywords:** Genetics research, Genome-wide association studies, Obesity

## Abstract

Obesity is a major risk factor for many common diseases and has a substantial heritable component. To identify new genetic determinants, we performed exome-sequence analyses for adult body mass index (BMI) in up to 587,027 individuals. We identified rare loss-of-function variants in two genes (*BSN* and *APBA1*) with effects substantially larger than those of well-established obesity genes such as *MC4R*. In contrast to most other obesity-related genes, rare variants in *BSN* and *APBA1* were not associated with normal variation in childhood adiposity. Furthermore, *BSN* protein-truncating variants (PTVs) magnified the influence of common genetic variants associated with BMI, with a common variant polygenic score exhibiting an effect twice as large in *BSN* PTV carriers than in noncarriers. Finally, we explored the plasma proteomic signatures of *BSN* PTV carriers as well as the functional consequences of *BSN* deletion in human induced pluripotent stem cell-derived hypothalamic neurons. Collectively, our findings implicate degenerative processes in synaptic function in the etiology of adult-onset obesity.

## Abstract (web summary)

Analyses of whole-exome sequencing data identify rare loss-of-function variants in *BSN* associated with adult-onset obesity, type 2 diabetes and fatty liver disease, with stronger effect sizes than those observed for variants in known obesity risk genes such as *MC4R.*

## Main

Over 1 billion people worldwide live with obesity, a global health challenge that is rapidly increasing in scale^1,2^. Obesity is the second leading cause of preventable death, increasing the risk of diseases such as type 2 diabetes (T2D), cardiovascular disease and cancer^1,3^. Understanding the full range of social, psychological and biological determinants of energy intake and expenditure will be key to tackling this epidemic. Early studies in mice highlighted the role of the leptin−melanocortin pathway in appetite and body weight regulation^4^, which led to candidate gene sequencing studies of individuals with severe early-onset obesity. These studies identified rare loss-of-function mutations in key components of this pathway as causes of severe early-onset obesity^5^, the most common of which affect the melanocortin 4 receptor (*MC4R*)^6,7^. In parallel, using a ‘hypothesis-free’ approach, large-scale population-based genome-wide association studies (GWAS) have identified hundreds of common genetic variants associated with body mass index (BMI) in adults^8^. These variants are mostly noncoding and are enriched near genes expressed in the brain^9^. Individually, the effect of each variant is small, and cumulatively, the ~1,000 common variants identified to date explain only ~6% of the population variance in BMI^8^.

The recent emergence of whole-exome sequencing (WES) data at the population scale has enabled exome-wide association studies (ExWAS), leading to a convergence of common and rare variant discoveries. In a landmark study, Akbari et al. used WES data from ~640,000 individuals to identify rare protein-coding variants in 16 genes associated with BMI^10^. These included genes with established roles in weight regulation (*MC4R*, *GIPR* and *PCSK1*) in addition to new targets, such as *GPR75*, in which loss-of-function mutations are protective against obesity in humans and mice^10^.

The current study was an ExWAS for BMI using WES data from 419,668 UK Biobank participants. Although this represents a subset of the exomes previously reported by Akbari et al.^10^, we were motivated by recent work demonstrating that, in the context of gene-burden analysis^11^, the various choices around how one defines a qualifying rare variant can highlight biologically relevant genes at exome-wide significance missed using alternative definitions^12^. Consistent with this, our approach identified new rare variant associations with *BSN* and *APBA1*, which we replicated in independent WES data from 167,359 individuals of predominantly non-European genetic ancestry. The rare protein-truncating variants (PTVs) detected in *BSN* and *APBA1* have larger effects than other previously reported ExWAS genes^10^, and our findings collectively suggest emerging roles for neurodevelopment, neurogenesis and altered neuronal oxidative phosphorylation in the etiology of obesity.

## Results

### Exome-sequence analysis identifies rare alleles associated with BMI

To identify rare variants associated with adult BMI, we performed an ExWAS using genotype and phenotype data from 419,668 individuals of European ancestry from UK Biobank^13^. Individual gene-burden tests were performed by collapsing rare (minor allele frequency (MAF) < 0.1%) genetic variants across 18,658 protein-coding genes. We tested three categories of variants based on their predicted functional impact: high-confidence (HC) PTVs and two overlapping missense masks that used a REVEL^14^ score threshold of 0.5 or 0.7. This yielded a total of 37,691 gene tests with at least 30 informative rare allele carriers, corresponding to a multiple-test-corrected statistical significance threshold of *P* < 1.33 × 10^−6^ (0.05/37,691).

Genetic association testing was performed using BOLT-LMM^15^, which identified a total of nine genes that met the threshold for significant association with adult BMI (Supplementary Table 1). Our gene-burden ExWAS appeared to be statistically well calibrated, as indicated by low exome-wide test statistic inflation (*λ*_GC_ = 1.05−1.15) and by the absence of significant associations with any synonymous variant masks (Supplementary Figs. 1 and 2). Five of our identified associations were previously reported: PTVs in *MC4R*, *UBR2*, *KIAA1109*, *SLTM* and *PCSK1* (ref. ^10^). At the other four genes, heterozygous PTVs conferred higher risk for increased adult BMI: *BSN* (effect = 3.05 kg m^−2^, standard error (s.e.) = 0.54, *P* = 2 × 10^−8^, carrier *n* = 65), *TOX4* (effect = 3.61 kg m^−2^, s.e. = 0.71, *P* = 3.1 × 10^−7^, carrier *n* = 39), *APBA1* (effect = 2.08 kg m^−2^, s.e. = 0.42, *P* = 6.1 × 10^−7^, carrier *n* = 111) and *ATP13A1* (effect = 1.82 kg m^−2^, s.e.m. = 0.37, *P* = 1.1 × 10^−6^, carrier *n* = 139). For two of these genes, *BSN* and *ATP13A1*, we also found supporting evidence from common genetic variants at the same locus associated with BMI (Supplementary Fig. 3): noncoding alleles ~200 kb upstream of *BSN* (rs9843653, MAF = 0.49, *β* = −0.13 kg m^−2^, *P* = 9.5 × 10^−46^) and 400 kb upstream of *ATP13A1* (rs72999063, MAF = 0.16, *β* = 0.09 kg m^−2^, *P* = 3.2 × 10^−13^; Supplementary Table 2). These GWAS signals were also associated with blood RNA expression levels of *BSN* and *ATP13A1*, respectively^16^ (Supplementary Table 2), and the BMI associations were replicated in independent GWAS data from the GIANT consortium^9^ (Supplementary Fig. 4 and Supplementary Table 2). We found no evidence of rare variant associations with BMI for any other genes at these GWAS loci (Supplementary Table 3).

**Fig. 1:**
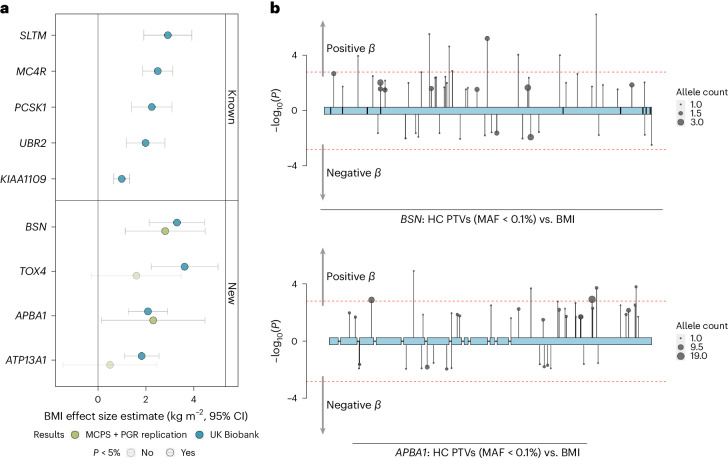
Discovery and replication of new rare variant associations with BMI. **a**, Discovery analyses were conducted in UK Biobank (*n* = 419,668) and replication was conducted in individuals from the MCPS and PGR study (*n* = 167,359). The means of the effect size estimates are presented with 95% CIs and were converted to kg m^−2^. Extended data can be found in Supplementary Tables 1 and 4. **b**, Variant-level results from the BOLT-LMM algorithm using a linear mixed model for association of HC PTVs in *BSN* and *APBA1* with BMI. The *y* axis shows trait-increasing effects with −log_10_(*P*) and trait-decreasing effects with log_10_(*P*). The dashed lines denote a nominal significance threshold of *P* < 0.05. Statistics used to generate these plots are provided as source data. Source data

We aimed to replicate our four new gene-burden rare variant associations in independent WES data from 167,359 individuals of predominantly non-European ancestry from the Mexico City Prospective Study (MCPS)^17,18^ and the Pakistan Genomic Resource (PGR) study (Fig. 1 and Supplementary Table 4). We observed supportive evidence for two of the four new genes identified above: for 32 *BSN* PTV carriers the mean BMI was 2.8 kg m^−2^ (s.e. = 0.84, *P* = 9.4 × 10^−4^) higher than for noncarriers, and for 20 *APBA1* PTV carriers the mean BMI was 2.33 kg m^−2^ (s.e. = 1.05, *P* = 0.03) higher. Although the replication sample was smaller than the UK Biobank sample and evidence for replication at *APBA1* was only nominally significant, these effect sizes were remarkably similar to those observed in UK Biobank (3.05 kg m^−2^ and 2.08 kg m^−2^ for *BSN* and *APBA1*, respectively).

**Fig. 2:**
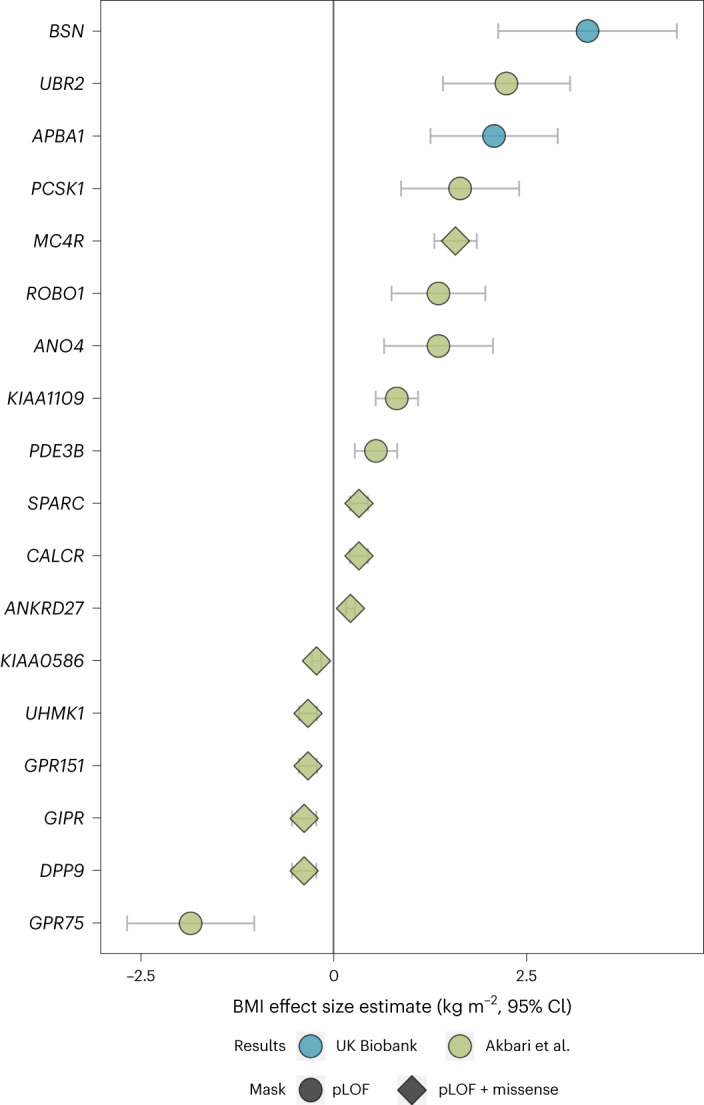
Comparison of effects between replicated associations and previously reported associations. The means of the effect size estimates on BMI are presented with 95% CIs and are based on only UK Biobank participants (*n* = 419,668). The statistics used to generate this plot are provided as source data. pLOF, predicted loss of function. Source data

**Fig. 3:**
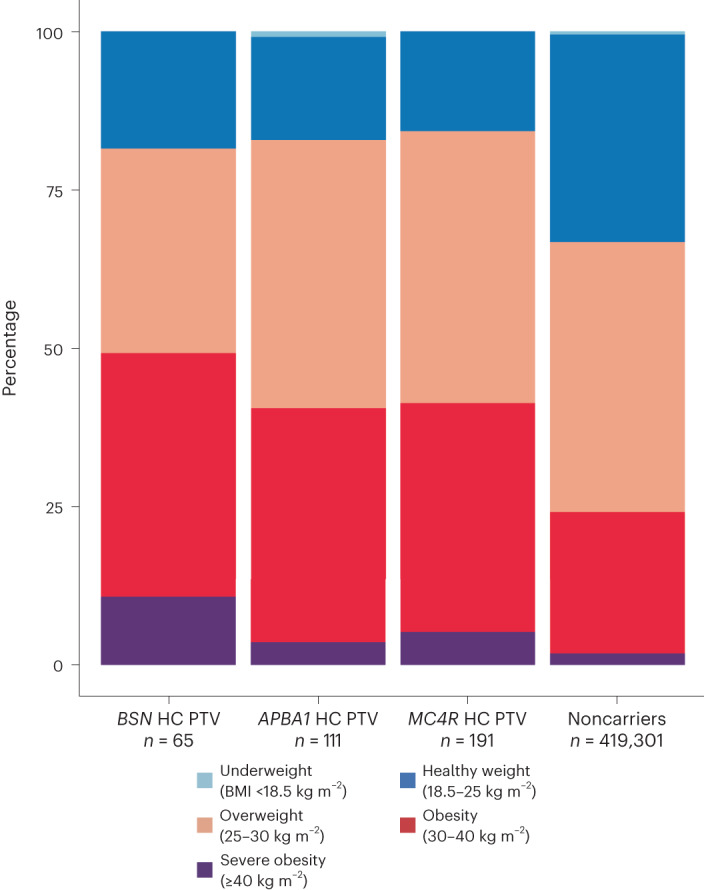
Distribution of BMI categories for carriers and noncarriers of *BSN*, *APBA1* or *MC4R* HC PTVs. The BMI categories appear according to guidance from the World Health Organization. The statistics used to generate this plot are provided as source data. Source data

The effect of *BSN* on BMI was larger than that of any previously reported ExWAS gene (Fig. 2) and substantially increased the risk of obesity (BMI > 30 kg m^−2^) in UK Biobank (*BSN*: odds ratio (OR) = 3.04 (95% confidence interval (CI), 1.87−4.94), *P* = 7.7 × 10^−6^, 49% case prevalence; *APBA1*: OR = 2.14 (1.46−3.13), *P* = 8.5 × 10^−5^, 41% case prevalence) and for *BSN* also increased the risk of severe obesity (BMI > 40 kg m^−2^) (OR = 6.61 (3.01−14.55), *P* = 2.6 × 10^−6^, 11% case prevalence) although this was not the case for *APBA1* (OR = 1.91 (0.70−5.19), *P* = 0.20, 4% case prevalence; Fig. 3). Association statistics for individual variants in *BSN* and *APBA1* in UK Biobank are shown in Fig. 1b and Supplementary Table 5. The gene-level associations of *BSN* and *APBA1* with BMI were not driven by single HC PTVs (Supplementary Table 6), and carriers appeared to be geographically dispersed across the UK (Supplementary Fig. 5).

In a case−cohort study that included the Severe Childhood-Onset Obesity Project (SCOOP) and the INTERVAL Study (INTERVAL), we identified an excess of *BSN* PTV carriers among patients affected by severe early-onset obesity (3/927 cases; p.Arg1276*, p.Arg1787*, p.Arg2925*; Supplementary Table 4) compared to the control cohort (1/4,057; OR = 13 (1.05−686), *P*_exact_ = 0.02). Furthermore, the one PTV found among controls (p.Trp3926*) is located at the final amino acid of the *BSN*-encoded protein bassoon and is therefore unlikely to affect its function (*P*_exact_ = 0.006, when excluding p.Trp3926*).

### Phenotypic characterization of *BSN* and *APBA1* rare allele carriers

We next sought to understand the broader phenotypic profile of carriers of PTVs in *BSN* and *APBA1*. In UK Biobank, these genes showed diverse associations with body composition, with higher fat and lean mass across body compartments (Supplementary Table 7), but showed no association with adult height (*P* > 0.05) or waist-to-hip ratio adjusted for BMI (*P* > 0.05). In contrast to almost all previously reported obesity-associated genes, neither *BSN* nor *APBA1* showed any association with childhood body size or puberty timing (*P* > 0.05), suggesting adult-onset effects on body weight based on the phenotypes available in UK Biobank. In UK Biobank, carriers of PTVs in *BSN* also had a higher risk of T2D (OR = 3.03, (1.60−5.76), *P* = 7.1 × 10^−4^, 18% case prevalence)—an effect size comparable to those of previously reported rare variant associations for T2D^19,20^. A broader phenome-wide analysis across 11,693 traits revealed a number of other associations (Supplementary Table 8); notably, *BSN* PTV carriers had a substantially higher risk of nonalcoholic fatty liver disease, as defined by a fatty liver index of ≥60 (ref. ^21^) or a hepatic steatosis index of >36 (ref. ^22^), compared to noncarriers (OR = 3.73 (2.26−6.16), *P* = 8.4 × 10^−7^, 45% case prevalence).

### *BSN* carrier status magnifies the effect of common genetic variants

Previous studies have reported that common BMI-associated alleles increased the penetrance of obesity in rare allele carriers in an additive model^10^. To evaluate this for *BSN* and *APBA1*, we created a common variant polygenic score (PGS) in UK Biobank, using individual variant effect estimates obtained from independent GIANT consortium GWAS data^9^. By testing the multiplicative interaction between the PGS and rare variant carrier status on BMI in a linear regression model, we observed significant effect modification by *BSN* PTVs (interaction *P* = 0.01; Supplementary Fig. 6), but not *APBA1* PTVs (*P* = 0.22). In carriers of *BSN* PTVs, the effect size of the PGS on BMI was double (0.6 s.d. increase in BMI per unit increase in PGS, equivalent to 2.9 kg m^−2^) that in noncarriers (0.3 s.d., equivalent to 1.4 kg m^−2^).

### Evaluating the impact of *BSN* and *APBA1* functions on the plasma proteome

To explore the putative biological mechanisms through which *BSN* and *APBA1* might exert their effects, we first characterized the plasma proteomic signature of PTV carriers using Olink data on 1,463 circulating proteins available in ~50,000 UK Biobank participants^23,24^. Using the available proteomics data, we identified 6 and 17 PTV carriers for *BSN* and *APBA1*, respectively. No changes in plasma protein levels were associated with *APBA1* carrier status after multiple-test correction (*P* < 3.42 × 10^−5^ (0.05/1,463)); however, *BSN* PTV carriers had higher levels of lymphotoxin alpha (LTα, previously known as TNFβ) than noncarriers (effect = 1.07, s.e. = 0.183, *P* = 5.3 × 10^−9^) (Supplementary Table 9). Furthermore, circulating LTα levels were positively associated with BMI (increase of 1.18 kg m^−2^ in BMI per 1 s.d. increase in LTα concentration, *P* = 7.6 × 10^−122^), and common genetic variants at the *LTA* locus were associated with BMI (rs3130048, MAF = 0.72, *β* = −0.10 kg m^−2^ per allele, *P* = 1.10 × 10^−23^). We repeated these analyses using the common BMI-associated variant (rs9843653) at *BSN* and identified 23 associated proteins, the most significant of which was semaphorin-3F (−0.03 s.d. per BMI-increasing allele, *P* = 6.7 × 10^−45^), a member of the semaphorin family that has been previously implicated in obesity etiology^25^. In total, 10 of the genes encoding these 24 proteins (including *SEMA3F* and *LTA*) were also implicated by common variant signals for BMI (Supplementary Table 10).

### Differential gene expression in *BSN*^+/−^ hypothalamic neurons

Finally, we explored the functional consequences of deleting *BSN*, which is highly expressed in the brain, by generating CRISPR−Cas9-edited human induced pluripotent stem cell-derived hypothalamic neurons heterozygous for the *BSN* p.Leu400Trpfs*114 PTV (*BSN*^+/−^) (Methods). On visual inspection, *BSN*^+/−^ cells showed no obvious morphological effect on neuronal differentiation (Supplementary Fig. 7). To assess transcriptional differences between *BSN*^+/−^ and wild-type cells, we performed single-nucleus RNA sequencing (snRNA-seq) in 61,016 hypothalamic neurons (32,198 *BSN*^+/−^, 28,818 wild type). We identified 18 distinct cell clusters, as shown via a uniform manifold approximation and projection plot (Supplementary Fig. 8; marker genes listed in Supplementary Table 11). Eight clusters were neurons (clusters 4, 5, 6, 9, 11, 13, 14 and 15; total *n* = 18,873) marked with *RBFOX3* (*NeuN*), *BSN* and the bassoon binding partner *PCLO* (Supplementary Fig. 8). Because *BSN* is universally expressed in neurons, we combined expression data across all eight neuronal clusters in the differential gene expression analysis and performed pathway enrichment analyses to examine the possible global consequences of *BSN*^+/−^. Differential expression analyses revealed 778 genes (defined by *P* < 0.05 and log_2_(fold change (FC)) > 1 or < −1) (Supplementary Table 12), including downregulation of genes with reported roles in body weight regulation, such as *SEMA3C*^25^ and *APOE*^26,27^. The top enriched pathways included ‘neuroactive ligand-receptor interaction’ and ‘negative regulation of neurogenesis’, as well as ‘respiratory chain complex I (gamma subunit) mitochondrial’. Furthermore, when we examined the differential expression within individual clusters, *NTNG1* was downregulated (log_2_(FC) = −0.66 to −0.93, *P* < 0.05) in four of eight *BSN*^+/−^ populations (Supplementary Table 12). NTNG1 is closely associated with bassoon within the presynaptic active zone; it belongs to a class of synaptic adhesion molecules crucial for synaptic function^28^ and has a role in axon guidance in neurons^29^. Interestingly, common variants of *NTNG1* are associated with BMI^30,31^. Differentially expressed genes within cluster 13 were also enriched for common variant associations with BMI (Supplementary Tables 13 and 14), including associations in *APOE*, *DOC2A*, *COMT* and *GABPB2*. Taken together, these results highlight dysregulation of neurodevelopment, neurogenesis and neuronal oxidative phosphorylation as possible underlying mechanisms linking *BSN* deficiency to obesity (Supplementary Table 15).

## Discussion

We found that rare PTVs in *APBA1* and *BSN* were associated with a substantial increase in adult BMI and higher risks of obesity and severe obesity in adults. Rare PTVs in *BSN* were also associated with higher risks for T2D and nonalcoholic fatty liver disease. The associations with adult BMI were confirmed in independent cohorts and were also supported by mapping of common variant signals to whole-blood expression quantitative trait loci for *APBA1* and *BSN*. Rare PTVs in *BSN* were also found in three individuals with severe early-onset obesity; however, in UK Biobank, 65 *BSN* PTV carriers showed no difference in childhood adiposity-related traits compared to noncarriers. Therefore, *APBA1* and *BSN* appear to be among the few genetic determinants of predominantly adult-onset obesity. The recalled childhood adiposity trait in UK Biobank shows a high genetic correlation with measured childhood BMI^32^; however, we acknowledge that it may still be an insensitive measure and longitudinal studies are needed.

*APBA1* encodes a neuronal adaptor protein that interacts with amyloid precursor protein, encoded by the Alzheimer disease-associated *APP* gene. It has a putative role in signal transduction as a vesicular trafficking protein with the potential to couple synaptic vesicle exocytosis to neuronal cell adhesion^33^. *BSN* encodes bassoon, a scaffolding protein essential for organization of the presynaptic cytoskeleton and exocytosis-mediated neurotransmitter release^34^. *Bsn* knockout in mice reduces excitatory synaptic transmission because vesicles are unable to efficiently fuse with the synaptic membrane^35^. *BSN* is expressed primarily in the brain and is reportedly upregulated in the frontal lobes of patients with multiple system atrophy, a progressive neurodegenerative disease^36^. Furthermore, rare predicted-damaging missense mutations in *BSN* have been reported in four patients with progressive supranuclear palsy-like syndrome with features of multiple system atrophy and Alzheimer disease^37^. The links identified here with predominantly adult-onset obesity may be consistent with the putative roles of *APBA1* and *BSN* in aging-related neurosecretory vesicle dysfunction and neurodegeneration. Therefore, we posit that adult obesity could result from some form of subtle age-dependent degeneration in primary appetitive regulatory pathways.

Previous studies have reported additive effects of common and rare susceptibility alleles on BMI^10^, but there is no evidence for epistatic interactions that are indicative of biological interactions. Notably, we found that carriers of rare PTVs in *BSN* showed enhanced susceptibility to the influence of a common variant PGS for adult BMI. The mechanistic basis for this statistical interaction is unclear. However, as the common genetic susceptibility to obesity is thought to act predominantly via central regulation of food intake^9,38^, we hypothesize that *BSN* may have widespread involvement in neurodevelopment and neurogenesis, with *BSN* variants leading to increased appetitive drive. We propose that future studies explore the impact of *BSN* PTVs on primary appetitive regulatory pathways across the life course.

The associations identified with rare PTVs in *APBA1* and *BSN* were not highlighted in previous ExWAS analyses using overlapping data. We acknowledge the differences between such studies in relation to variant quality control and the thresholds used for in silico functional prediction. We posit that standardization in this field would be premature. Instead, studies should clearly detail their analytical approaches and seek replication and other forms of confirmation.

In conclusion, rare genetic disruptions of *APBA1* and *BSN* have larger impacts on adult BMI and obesity risk than heterozygous disruptions of any previously described obesity risk gene. Rare PTVs in *APBA1* and *BSN* appear to preferentially confer risk of adult-onset obesity, which we propose might be due to widespread dysregulation of neurodevelopment, neurogenesis and neuronal oxidative phosphorylation in neurons within the central feeding circuitry.

## Methods

### Ethics

Our research complies with all relevant ethical regulations. All studies included in this research were approved by the relevant board or committee. UK Biobank has approval from the North West Multi-centre Research Ethics Committee (REC reference 13/NW/0157) as a Research Tissue Bank (RTB) approval, and informed consent was provided by each participant. This approval means that researchers do not require separate ethical clearance and can operate under RTB approval. This RTB approval was granted initially in 2011 and is renewed every 5 years; hence, UK Biobank successfully renewed approval in 2016 and 2021. The MCPS was approved by the Mexican Ministry of Health, the Mexican National Council for Science and Technology and the University of Oxford. The PGR study was approved by the institutional review board at the Center for Non-Communicable Diseases (IRB: 00007048, IORG0005843, FWAS00014490) and all participants provided informed consent. The SCOOP cohort was approved by the Multi-regional Ethics Committee and the Cambridge Local Research Ethics Committee (MREC 97/21 and REC number 03/103). Participants (or parents for individuals <16 years old) provided written informed consent; minors provided oral consent. The INTERVAL study received ethics committee approval from the National Research Ethics Service Committee (11/EE/0538), and all participants provided informed consent before joining the study.

### UK Biobank data processing and quality control

We used the same processing strategies as those outlined in our previous paper to analyze the WES data and perform quality control steps^19^. We queried WES data from 454,787 individuals in UK Biobank^39^, excluding those with excess heterozygosity, those with autosomal variant missingness on genotyping arrays of ≥5%, or those not included in the subset of phased samples as defined by Bycroft et al.^13^.

WES data were stored as population-level variant call format (VCF) files, aligned to GRCh38 and accessed through the UK Biobank Research Analysis Platform (RAP). In addition to the quality control measures already applied to the released data, as described by Backman et al.^39^, we conducted several additional quality control procedures. First, we used ‘bcftools v1.14 norm’^40^ to split the multiallelic sites and left-correct and normalize indels. Next, we filtered out variants that failed our quality control criteria, including those with: (1) read depth of <7; (2) genotype quality of <20; and (3) binomial test *P* value for alternative allele reads versus reference allele reads of ≤0.001 for heterozygous genotypes. For indel genotypes, we kept only variants with read depth of ≥10 and genotype quality of ≥20. Variants that failed quality control criteria were marked as missing (that is, ./.). After filtering, variants where more than 50% of the genotypes were missing were excluded from downstream analyses^19^.

The remaining variants underwent annotation using Ensembl Variant Effect Predictor (VEP v104)^41^ with the ‘-everything’ flag and additional plugins for REVEL^14^, CADD^42^ and LOFTEE^43^. For each variant, a single Ensembl transcript was prioritized on the basis of whether the annotated transcript was protein-coding, MANE select v0.97 (ref. ^44^) or the VEP canonical transcript. The individual consequence for each variant was then prioritized on the basis of severity as defined by VEP. Stop-gained, splice acceptor and splice donor variants were merged into a combined PTV category, while annotations for missense and synonymous variants were adopted directly from VEP. We included only variants on autosomes and the X chromosome that were within Ensembl protein-coding transcripts and transcripts included in the UK Biobank WES assay in our downstream analysis.

Our analyses focused primarily on individuals of European genetic ancestry, and we excluded those who withdrew consent from the study, resulting in a final cohort of 419,668 individuals.

### Exome-wide gene-burden testing in UK Biobank

We used BOLT-LMM v2.3.6 (ref. ^15^) as our primary analytical tool to conduct the gene-burden test. To run BOLT-LMM, we first queried a set of genotypes with minor allele count (MAC) > 100, which was derived from the genotyping arrays for the individuals with the WES data to build the null model. To accommodate BOLT-LMM’s requirement for imputed genotyping data rather than per-gene carrier status, we developed dummy genotype files in which each gene was represented by a single variant. We then coded individuals with a qualifying variant within a gene as heterozygous, regardless of the total number of variants they carried in that gene. We then created dummy genotypes for the HC PTVs with MAF < 0.1% as defined by LOFTEE, missense variants with REVEL > 0.5 and missense variants with REVEL > 0.7. We then used BOLT-LMM to analyze phenotypes using default parameters, except for the inclusion of the ‘lmmInfOnly’ flag. In addition to the dummy genotypes, we included all individual markers in the WES data to generate association test statistics for individual variants. We used age, age^2^, sex and the first ten principal components (PCs) as calculated by Bycroft et al.^13^ and the WES release batch (50k, 200k, 450k) as covariates.

To check whether there was a single variant driving the association, we performed a leave-one-out analysis for *BSN* and *APBA1* using linear regression in R v3.6.3 by dropping the HC PTVs contained in our analysis one by one. In addition, we also checked the geographic distribution of *APBA1* and *BSN* HC PTV carriers.

### Replication of findings in two independent non-European cohorts

We sought replication of our findings for the four new genes in two independent predominantly non-European exome-sequenced cohorts: the MCPS and the PGR study.

MCPS is a cohort study of 159,755 adults of predominantly admixed American ancestry. Participants aged 35 years or older were recruited between 1998 and 2004 from two adjacent urban districts of Mexico City. Phenotypic data were recorded during household visits, including height, weight, and waist and hip circumferences. Disease history was self-reported at baseline, and the participants were linked to Mexican national mortality records. The cohort has been described in detail elsewhere^17,18^.

The PGR study has been recruiting participants aged 15−100 years as cases or controls via clinical audits for specific conditions since 2005 from over 40 centers around Pakistan. Participants were recruited from clinics treating patients with cardiometabolic, inflammatory, respiratory or ophthalmological conditions. Information on lifestyle habits, medical and medication history, family history of diseases, exposure to smoking and tobacco consumption, physical activity, dietary habits, anthropometry, basic blood biochemistry and electrocardiogram traits was recorded during clinic visits. DNA, serum, plasma and whole blood samples were also collected from all study participants.

Exome sequencing data for 141,046 MCPS and 37,800 PGR participants were generated at the Regeneron Genetics Center and passed Regeneron’s initial quality control, which included identifying sex discordance, contamination, unresolved duplicate sequences and discordance with microarray genotype data for MCPS. Genomic DNA was subjected to paired-end 75-bp WES at Regeneron Pharmaceuticals using the IDT xGen v1 capture kit on the NovaSeq 6000 platform. Conversion of sequencing data in BCL format to FASTQ format and the assignments of paired-end sequence reads to samples were based on 10-base barcodes, using bcl2fastq v2.19.0.

These exome sequences were processed at AstraZeneca from their unaligned FASTQ state. A custom-built Amazon Web Services cloud computing platform running Illumina DRAGEN Bio-IT Platform Germline Pipeline v3.0.7 was used to align the reads to the GRCh38 genome reference and perform single-nucleotide variant (SNV) and insertion and deletion (indel) calling. SNVs and indels were annotated using SnpEff v4.3 (ref. ^45^) against Ensembl Build 38.92. All variants were additionally annotated with their gnomAD MAFs (gnomAD v2.1.1 mapped to GRCh38)^43^.

To further apply quality control to the sequence data, all MCPS and PGR exomes underwent a second screening using AstraZeneca’s bioinformatics pipeline, which has been described in detail previously^46^. Briefly, we excluded from the analysis sequences that had a VerifyBamID freemix (contamination) level of more than 4%, those for which inferred karyotypic sex did not match self-reported gender or those for which less than 94.5% of the consensus coding sequence (CCDS release 22) achieved a minimum tenfold read depth. We further removed one individual from every pair of genetic duplicates or monozygotic twins with a kinship coefficient of >0.45. Kinship coefficients were estimated from exome genotypes using the kinship function from KING v2.2.3 (ref. ^47^). For the MCPS, we additionally excluded sequences with an average CCDS read depth of at least 2 s.d. below the mean. After the above quality control steps, 139,603 (99.0%) MCPS and 37,727 (99.3%) PGR exomes remained.

For the MCPS, we predicted the genetic ancestry of participants using PEDDY v0.4.2 (ref. ^48^), with 1000 Genomes Project sequences as population ref. ^49^, and retained individuals with a predicted probability of admixed American ancestry of ≥0.95 who were within 4 s.d. of the means for the top four PCs. In the PGR study, we retained individuals with a predicted probability of South Asian ancestry of ≥0.95 who were within 4 s.d. of the means for the top four PCs. Following ancestry filtering, 137,059 (97.2%) MCPS and 36,280 (95.5%) PGR exomes remained.

We assessed the association of BMI and weight quantitative traits with genotype at the four proposed new genes of interest using a previously described gene-level collapsing analysis framework implementing a PTV collapsing analysis model^46^. We classified variants as PTVs if they had been annotated by SnpEff as follows: exon_loss_variant, frameshift_variant, start_lost, stop_gained, stop_lost, splice_acceptor_variant, splice_donor_variant, gene_fusion, bidirectional_gene_fusion, rare_amino_acid_variant and transcript_ablation.

We applied MAF filters to target rare variants: MAF < 0.001 in gnomAD (overall and every population except OTH) and leave-one-out MAF < 0.001 among our combined case and control test cohort. For variants to qualify, they had to also meet the following quality control filters: minimum site coverage of 10×; annotation in CCDS transcripts (release 22); at least 80% alternative reads in homozygous genotypes; a percentage of alternative reads for heterozygous variants of ≥0.25 and ≤0.8; a binomial test of alternative allele proportion departure from 50% in the heterozygous state result of *P* > 1 ×10^−6^; GQ of ≥20; FS of ≤200 (indels) or ≤60 (SNVs); MQ of ≥40; QUAL of ≥30; read position rank sum score of ≥−2; MQRS of ≥−8; DRAGEN variant status = PASS; and test cohort carrier quality control failure of < 0.5%. If the variant was observed in gnomAD exomes, we also applied the following filters: variant site achieved tenfold coverage in ≥25% of gnomAD exomes; variant site achieved exome *z*-score of ≥−2.0; exome MQ of ≥30; and random forest probability that the given variant is a true SNV or indel of >0.02 and >0.01, respectively^50^.

For the quantitative traits and for each gene, the difference in mean between the carriers and noncarriers of PTVs was determined by fitting a linear regression model, correcting for age and sex. In addition to calculating individual statistics for the MCPS and the PGR study, we also meta-analyzed the individual study effect sizes to generate a combined replication statistic using an inverse variance-weighted fixed-effect meta-analysis using the rma.uni() function from the metafor package v3.8-1 (ref. ^51^) in R v3.6.3.

### *BSN* PTV carriers in the SCOOP−INTERVAL case−cohort study

To test whether there was an association between pLOF variants in the *BSN* gene and severe early-onset obesity, we studied 927 exomes from white British participants with severe early-onset obesity recruited to the Genetics of Obesity Study (GOOS) (SCOOP cohort) and 4,057 control exomes from the INTERVAL cohort of UK blood donors. SCOOP comprises UK patients with severe obesity (BMI more than 3 s.d. above the mean for age and sex) of early onset (<10 years) recruited to the GOOS. Exome sequencing in a subset of people of white British ancestry (the SCOOP cohort) was performed as described previously^52–54^. INTERVAL comprises predominantly healthy blood donors in the UK^55^ (https://www.intervalstudy.org.uk).

SCOOP and INTERVAL variants were joint-called and filtered for variant-level and sample-level quality control, as previously described^52^. A total of 927 cases (SCOOP) and 4,057 controls (INTERVAL) passed the quality control filters^53^. After splitting multiallelic variants and left normalizing, we annotated variants using VEP with Ensembl v96 (GRCh37) and identified high-impact variants (predicted protein-truncating, null or splice-disrupting) in the gene *BSN* (transcript ENST00000296452) using VEP IMPACT=‘HIGH’. This definition includes stop-gain variants (SNVs resulting in stop codons), frameshifts and splice donor/acceptor variants. We verified that the predicted consequences and stop codon positions were maintained in the latest minor version of the transcript (ENST00000296452.5, NM_003458.4) using VEP v110 after lifting over to GRCh38. Missense variants were detected in almost all *BSN* exons among SCOOP exomes (7/10 coding exons) and INTERVAL exomes (8/10 coding exons), suggesting that *BSN* stop-gain detection rates in cases and controls are unlikely to be driven by differential read coverage within the *BSN* gene.

The one PTV identified in INTERVAL (p.Trp3926*) is located at the final amino acid of the bassoon protein and is therefore unlikely to affect expression levels (note that the LOFTEE in silico stop-gain filter for low-confidence loss of function based on the ‘50-bp rule’ does not apply to the *BSN* gene because the termination codon is itself >55 bp from the final exon−exon boundary^56^). After excluding this variant on the basis of low confidence for loss of function, we performed a nested gene-burden analysis on the remaining three variants: *n* = 3 pLOF carriers in SCOOP and *n* = 0 carriers in INTERVAL controls (OR (95% CI) = inf (1.8−inf), *P* = 0.006, Fisher’s exact test; adding +0.5 to each cell, OR = 31). Studies in vitro are required to establish the effect of each stop-gain variant on bassoon protein expression levels and localization.

### Phenome-wide analysis in UK Biobank

We included binary and quantitative traits made available in the June 2022 UK Biobank data release, harmonizing the phenotype data as previously described^46^. This resulted in 11,690 phenotypes for analysis, which are available on https://azphewas.com. On the basis of clinical relevance, we derived three additional phenotypes.

For UK Biobank phenome-wide analyses of the four putatively new genes, the same data generation and quality control processes described for the MCPS and PGR study were applied to UK Biobank exomes. Following the Regeneron and AstraZeneca quality control steps, 445,570 UK Biobank exomes remained. The phenome-wide analysis was performed in UK Biobank participants of predominantly European descent, whom we identified based on a PEDDY-derived predicted probability of European ancestry of ≥0.95 and were within 4 s.d. of the means for the top four PCs. On the basis of predicted ancestry pruning, 419,391 UK Biobank exomes were included in the phenome-wide analyses of the four prioritized genes.

As described previously, we assessed the association of the 11,693 phenotypes with genotypes at the four genes of interest, using a PTV collapsing analysis model^46^, and classifying variants as PTVs using the same SnpEff definitions as described for the MCPS and PGR analyses. For variants to qualify for inclusion in the model, we applied the same MAF and quality control filters used in the MCPS and PGR analyses, with the exception that due to the larger sample size of UK Biobank, only <0.01% of the test cohort carriers were permitted to fail quality control.

### Association testing for other anthropometric phenotypes and protein expression levels

We ran association tests of *APBA1* and *BSN* HC PTV carriers and carriers of a BMI-associated common variant (rs9843653) at the *BSN* locus with a list of anthropometric phenotypes available in UK Biobank using R v3.6.3 (Supplementary Table 5), including the same covariates we used in our exome-wide gene-burden tests. We acquired normalized protein expression data generated by the Olink platform from the UK Biobank RAP^23,24^. The detailed Olink proteomics assay, data processing and quality control were described by Sun et al.^23^. For the association tests of *APBA1* and *BSN* PTV carriers and BMI-associated common variant (rs9843653) at the *BSN* locus carriers with expression levels for 1,463 proteins, we added age^2^, age × sex, age^2^ × sex, Olink batch, UK Biobank center, UK Biobank genetic array, number of proteins measured and the first 20 genetic PCs as covariates, as suggested by Sun et al.^23^. We chose the Bonferroni-corrected *P* value (*P* < 3.42 × 10^−5^ (0.05/1,463)) as the threshold for significance.

### BMI GWAS lookup and downstream analyses

Identified genes were queried for proximal BMI GWAS signals, using data from UK Biobank, for signals within 500 kb upstream of the gene’s start site to 500 kb downstream of the gene’s end site. Such signals were further replicated in an independent BMI GWAS^9^.

We also performed colocalization tests, using the approximate Bayes factor method in R v4.0.2 using the package ‘coloc’ v5.1.0 and blood gene expression data from the eQTLGen study^16^. Genomic regions were defined as the regions ±500 kb around each gene, and loci exhibiting an *H*4 posterior probability of >0.5 were considered to show evidence of colocalization.

Finally, we used the GWAS data to calculate gene-level common variant associations, using MAGMA v1.09 (ref. ^57^). To do this, we used all common but nonsynonymous (coding) variants within a given gene. Gene-level scores were further collapsed into pathway-level associations where appropriate.

### Interaction effect between the PGS and PTV carrier status

To examine whether there is an interaction effect between PTV carrier status for *BSN* and *APBA1* and the PGS, we included an interaction term between the PGS and the carrier status for *BSN* and *APBA1* PTVs in a linear regression model adjusted for sex, age and age^2^, and the first 10 PCs.

The PGS was constructed for 419,581 individuals of white European ancestry who had both genotype and exome sequencing data and a BMI record in UK Biobank. We used summary statistics of BMI from Locke et al.^9^, which included samples not in UK Biobank. Data were downloaded from the GIANT consortium. The summary statistics included 2,113,400 single-nucleotide polymorphisms (SNPs) with at least 500,000 samples in a cohort of 322,154 participants of European ancestry. For the genotype data of UK Biobank participants, a light quality check procedure was applied, where SNPs were removed if they had a MAF of <0.1%, Hardy−Weinberg equilibrium *P* < 1 × 10^-6^ or more than 10% missingness. In addition, SNPs that were mismatched with those in the summary statistics (with the same rsID but different chromosomes or positions) were excluded. We used the package ‘lassosum’ v4.0.5 (ref. ^58^) in R v3.6.0 to construct the PGS. The *R*^2^ of the model including the PGS regressed on rank-based inverse normal-transformed BMI and adjusted for sex, age and age^2^, and the first 10 PCs as covariates was 11%.

### Cellular work and single-cell analyses

A detailed description of the methods used in cellular work and single-cell analyses can be found in the Supplementary Note.

### Reporting summary

Further information on research design is available in the Nature Portfolio Reporting Summary linked to this article.

## Online content

Any methods, additional references, Nature Portfolio reporting summaries, source data, extended data, supplementary information, acknowledgements, peer review information; details of author contributions and competing interests; and statements of data and code availability are available at 10.1038/s41588-024-01694-x.

### Supplementary information

Supplementary information Supplementary Notes and Figs. 1−8.

Reporting Summary

Supplementary Tables Supplementary Tables 1−16.

### Source data

Source Data Fig. 1 Statistical source data.

Source Data Fig. 2 Statistical source data.

Source Data Fig. 3 Statistical source data.

## Data Availability

The UK Biobank phenotype and WES data described here are publicly available to registered researchers through the UK Biobank data access protocol. Information about registration for access to the data is available at https://www.ukbiobank.ac.uk/enable-your-research/apply-for-access. Data for this study were obtained under resource applications 26041 and 9905. The MCPS welcomes open-access and collaboration data requests from bona fide researchers. For more details on accessibility, the study’s data and sample sharing policy can be downloaded (in English or Spanish) from https://www.ctsu.ox.ac.uk/research/mcps. Available study data can be examined in detail through the study’s Data Showcase, available at https://datashare.ndph.ox.ac.uk/mexico/. SCOOP and INTERVAL WES data are accessible from the European Genome-phenome Archive with accession numbers EGAS00001000124 (SCOOP) and EGAS00001000825 (INTERVAL). snRNA-seq data are available from the NCBI Gene Expression Omnibus (GEO), under accession number: GSE243112. Source data are provided with this paper.

## Code availability

The pipeline code for processing, filtering, annotating and burden testing UK Biobank WES data using the UK Biobank RAP is publicly available (https://github.com/mrcepid-rap)^59^. No custom code for analyzing the UK Biobank WES data was developed for this study. The analysis code for single-nucleus sequencing is available on GitHub (https://github.com/mariachukanova1/BSN_paper)^60^ and has been deposited on Zenodo at 10.5281/zenodo.10687754 (ref. ^61^).
