# Supplementary material for: Protein-truncating variants in BSN are associated with severe adult-onset obesity, type 2 diabetes and fatty liver disease
Source: Nat Genet. 2024 Apr 4;56(4):579–84. doi: 10.1038/s41588-024-01694-x (PMC11018524; doi:10.1038/s41588-024-01694-x)
Supplement: Supplementary file 1 — Supplementary Notes and Figs. 1−8. [file 41588_2024_1694_MOESM1_ESM.pdf]

nature genetics

Article https://doi.org/10.1038/s41588-024-01694-x

Protein-truncating variants in *BSN* are associated with severe adult-onset obesity, type 2 diabetes and fatty liver disease

In the format provided by the authors and unedited

Supplementary Notes

Cell lines and routine cell culture

The KOLF2.1J human-induced pluripotent stem cell line and its derivatives[1] were maintained on Geltrex (Thermo Fisher Scientific A1413202) coated plates in supplemented StemFlex media (Thermo Fisher Scientific A3349401) with daily medium changes. For passaging, the cells were washed with PBS and treated with TrypLE Express (Gibco, 12604021) at 37℃ for 3 min. The cells were re-suspended in StemFlex media supplemented with 10 µM ROCK Inhibitor Y-27632 dihydrochloride (Stemcell Technologies, 72304). ROCK inhibitor was removed the following day with growth medium without the Y-27632. Unless otherwise stated, cells were split at a 1:5 ratio. The absence of mycoplasma was confirmed using an EZ-PCR Mycoplasma Test Kit (Biological Industries, 20-700-20) following the manufacturer's instructions.

CRISPR-Cas9-mediated targeting of *BSN*

Two different small guide RNAs (sgRNA) with high predicted on-target and low predicted off-target activity were designed using CRISPick (https://portals.broadinstitute.org/gppx/crispick/public). For the production of sgRNAs the following constant oligonucleotide was used as a template for synthesis by *in vitro* transcriptionAAAGCACCGACTCGGTGCCACTTTTTCAAGTTGATAACGGACTAGCCTTAT TTTAACTTGCTATTTCTAGCTCTAAAAC 60. The following sequences were added to the variable sgRNA region sequences **(Supplementary Table 18):** SP6 promoter at the 5‘ end (ATTTAGGTGACACTATAGA) and at the 3’ end the following sequence, complementary to the 3’ end of the constant tracrRNA region (GTTTTAGAGCTAGAAATAGCAAG). In order to prevent frameshift from occurring, the first 2 nucleotides of the variable sgRNA region were left out. PAM sequence was not included in the sgRNA variable region. Resulting oligonucleotides, as well as the constant oligonucleotide, were then ordered from Sigma and resuspended in H_2O to a final concentration of 100 mM. Briefly, 1 µl of the constant oligo, 1 µl of the SP6 promoter-variable region oligonucleotide construct and 8 µl H2O were incubated for oligonucleotide annealing (95°C – 5 min; 95-85°C – decreasing by 2°C every sec; 85-25°C – decreasing by 0.1°C every sec; 4°C – forever). To fill in the single-stranded overhangs of the annealed oligonucleotides 10 µl of the BioTaq polymerase master mix (Bioline Bio-21040) was incubated with the oligos at 72°C for 15 minutes. Resulting oligonucleotides were purified using the Monarch PCR + DNA cleanup kit (New England

Biolabs T1030S) and eluted in 10 μl of DNA elution buffer from the kit. After that sgRNAs were made via *in vitro* transcription using the MEGAscript SP6 kit (Thermo Fisher, AM1330). The resulting sgRNAs were purified using the E.Z.N.A miRNA purification kit (Omega Bio-tek, R7034-01), eluted in RNase-free water, and stored at -80℃. Since sgRNAs vary in their efficacy, the relative cutting efficiencies of the two sgRNAs were tested in *in vitro* cleavage assays[2]. Briefly, amplicons of approximately 1000 base pairs, containing the mutation target site were generated. After that dilution series of Cas9 protein (10 μg/μl, 100 μg HiFi Cas9 nuclease V3, IDT 1081060) in assay buffer (200 mM KCl, 10 mM $MgCl_2$, 20 mM TrisCl, pH 8.0 in H_2O) was generated: 4 PCR tubes per sgRNA to be tested with 6 ml of Cas9 at 500 nM, 125 nM, 31 nM and 0 nM. After that 2 μl of the gDNA template and 2 μl of sgRNA at 200 ng/μl concentration were added to each tube to make a total 10 μl of the mix. Resulting concentrations of the Cas9 protein in the dilution series were: 300 nM, 75 nM, 19 nM and 0 nM. The reaction mix was then incubated at 37°C for 30 minutes. After that 1 μl of RNase A (ThermoFisher Scientific EN0531) was added to each reaction and the mix was incubated at 37°C for 10 minutes. After that 4 μl of reaction stop buffer (60 mM EDTA pH 8.0 (from stock UltraPure™ 0.5M EDTA, pH 8.0, Invitrogen, 15575-038), 2% SDS (Sigma 75746), 30% glycerol (from stock 100% glycerol Sigma G5516), dash of bromophenol blue (Sigma B5525)) was added to each reaction and the mix was incubated at 80°C for 10 min. The success of the cutting reactions was then then verified on a 1% agarose gel. We selected the sgRNAs that showed activity at the lowest Cas9 concentration for transfection into hPSC cells. After that the custom made sgRNA was ordered from (Integrated DNA Technologies Inc.). Single-stranded 100bp oligodeoxynucleotides (ssODN) templates (Integrated DNA Technologies Inc.) were constructed to contain target mutations in the middle and silent mutations within PAM sites. All sequences of the primers, sgRNA variable regions, ssODN donors used in the study are listed in **(Supplementary Table 16)**.

CRISPR-Cas9 ribonucleoprotein (RNP) complex-mediated editing in hESCs

To genetically edit the KOLF2.1J cells by homology-directed repair (HDR)[3,4], 3 µg purified sgRNA was mixed with 4 µg of recombinant Cas9 nuclease (IDT 1081060) for 45 min at room temperature to form stable ribonucleoprotein (RNP) complexes. The complex together with 1µl of 100 µM ssODN was then transferred to a 20µl single-cell suspension of 2 × 10^5 hESCs in P3 nucleofection solution and electroporated using Amaxa 4D-Nucleofector™ (Lonza) with program CA137. Transfected cells were seeded onto Geltrex-coated 24 well plates containing a pre-warmed StemFlex medium containing Revitacell (100x, Gibco

A2644501) and Penicillin/Streptavidin (ThermoFisher Scientific, 15140-122). HDR enhancer (IDT 1081072) was added to the cells at a 30μM final concentration for each well. The following day medium was changed to growth medium without Pen/Strep and Revitacell. To increase HDR efficiency, cells were cultured under cold shock conditions (32°C at 5% CO_2 in air atmosphere) for 48hr post transfection. Cells were given approximately 5-6 days to recover before single cells were then distributed into multiple Geltrex (1:40)-coated 96 well plates by an Aria-Fusion sorter with a 100 μm nozzle. After ~2 weeks, viable clonally-derived colonies were consolidated into duplicate 96 well plates to allow parallel cell cryopreservation and genomic DNA extraction[2,4].

Generation and sequencing of pooled amplicons

Genomic DNA (gDNA) was extracted using HotShot buffer[2]. Briefly, 50 μl per well of HotShot component 1 (25 mM NaOH, 0.2 mM EDTA in molecular biology grade H_2O) was added to the plates and the plates were incubates at 98°C for 30 minutes. Plates were then cooled to room temperature, condensation removed by briefly spinning the plates down and 50 μl per well of HotShot component 2 (50 mM Tris-HCl pH 8 in molecular biology grade H_2O) was added to the plates to produce the gDNA solution. The target regions were amplified from gDNA using locus-specific primers to generate amplicons approximately 150-200 bp in length. These "first-round" primers contained universal Fluidigm linker sequences at their 5'-end with the following sequences: Forward primer: 5'- acactgacgacatggttctaca -3', Reverse primer: 5'- tacggtagcagagacttggtct-3'. Specifically, 20 μl PCR reactions were set up in 96 well plates using 0.5U Phusion Hot Start II High-Fidelity DNA Polymerase (ThermoFisher Scientific, F-549L), 2 μl of extracted gDNA as template, 2 μl 5x GC buffer, 0.2 mM dNTPs, 2μM primers, and 3% DMSO, and run on the following programme: 98°C 30sec, followed by 24 cycles of (95°C 10 sec, 72°C 20 sec/ decreased by 0.5°C per cycle, 72°C 15 sec) than 12 cycles of (98°C 10sec, 60°C 30 sec, 72°C 15 sec) and 72°C 5 min. In the second round of PCR (indexing PCR), Fluidigm barcoding primers were attached to the amplicons to uniquely identify each clone. 2 μl linker PCR product diluted 1:10 was transferred to another 96-well PCR plate to perform this indexing PCR in 10 μl reactions containing 0.8 μM of Fluidigm barcoding primers, 2 μl 10x GC buffer, 0.2 mM dNTPs 3% DMSO and 0.5U Phusion Hot Start II polymerase. The PCR programme was 95°C 2 min, 16 cycles of (95°C 20 sec, 60°C 20 sec, 72°C 25 sec), 72°C 3 min. For sequencing library preparation, barcoded PCR products were combined in equal proportion based on estimation of band intensity on a 2% agarose gel, and the combined pool of PCR products was purified in a single tube using Ampure XP beads (Invitrogen 123.21D) at 1:1 (V/V) to the pooled sample and eluted in 25 μl of water according to the manufacturer's instructions. Library purity was

confirmed by nanodrop, and final library concentration was measured using the Agilent Bioanalyzer (High Sensitivity Kit, Agilent 5067-4626) and diluted to 20 nM. Pooled libraries could be combined with other library pools adjusted to 20 nM, and the resulting “superpool” volume was adjusted to a final volume of 20 µl before sequencing which is performed by the Genomics Core, Cancer Research UK Cambridge Institute. GenEditID platform[4] was used for identification of the *BSN* p.L400W*fs*114* heterozygous and wild type (WT) clones.

Hypothalamic neuron differentiation protocol

Gene edited *BSN* p.L400W*fs*114* heterozygous and WT clones were then differentiated into hypothalamic-like neurons[5,6]. Briefly, cells were cultured overnight on 10cm plate Geltrex coated plates (9.5 $x10^5$ cells/well for 6-well plates) in Stemflex™ with 10 µM ROCK inhibitor. Next day, neuroectoderm differentiation was initiated by dual SMAD inhibition using XAV939 (Stemgent 04-1946), LDN 193289 (Stemgent 04-0074) and SB 431542 (Sigma Aldrich S4317) and Wnt signaling inhibition using XAV939 (Stemgent 04-1946) in an in-house neural differentiation N2B27 medium[6]. From day 2 to day 7, cells were directed ‘towards ventral diencephalon’ with Sonic hedgehog activation, by the addition of Smoothened agonist SAG (1µM Thermo Fisher Scientific 56-666) and purmorphamine (PMC, 1 µM Thermo Fisher Scientific 54-022), with SMAD and Wnt inhibition molecules gradually replaced with N2 B27 medium changed every 2 days. At Day 8, the cells were switched into N2B27 with 5 µM DAPT (Sigma Aldrich D5942) to exit cell cycle. On Day 14, the cells were harvested with TrypLE™ supplemented with papain (Worthington LK003176) and re-plated onto laminin–coated 6-well plates at a density of $3x10^6$ cells per well in the presence of maturation medium containing brain-derived neurotrophic factor BDNF (10ng/ml, Sigma) containing N2B27. On day 16 media was changed to Synaptojuice 1 (N2B27, 10ng/ml BDNF, 2 µM PD0332991 (Sigma Aldrich, PZ0199), 5 µM DAPT, 370 µM $CaCl_2$ (Sigma Aldrich, 21115), 1 µM LM22A4 (Tocris, 4607), 2 µM CHIR99021 (Cell Guidance Systems, SM13), 300 µM GABA (Tocris, 0344), 10 µM NKH447 (Sigma Aldrich, N3290)). Cells were maintained in Synaptojuice 1 for a week before being changed to Synaptojuice 2 (N2B27, 10ng/ml BDNF, 2 µM, 370 µM $CaCl_2$ 1 µM LM22A4, 2 µM CHIR99021). Cells were then maintained in Synaptojuice 2 until day 36, with media renewal every second day throughout the differentiation and maturation period.

Single nucleus RNA-sequencing

On day 36, cells were dissociated using TrypLE™ and papain mixture, pelleted, and nuclei were isolated following a standardised 10x Genomics protocol for single nucleus RNA Sequencing (NucSeq)[7]. Sequencing libraries for the 6 (3 x wild type and 3 x *BSN* p.L400W*fs*114* Het) single-nuclei suspension samples were generated using 10X Genomics Chromium Single-Cell 3′ Reagent kits (Pleasanton, CA, USA, version 3.1) according to the manufacturer's menu. Briefly, nuclear suspensions were loaded onto the chromium chip along with gel beads, partitioning oil, and master mix to generate GEMs containing free RNA. RNA from lysed nuclei was reverse transcribed and cDNA was PCR amplified for 19 cycles. The amplified cDNA was used to generate a barcoded 3′ library according to the manufacturer's protocol, and paired-end sequencing was performed using an Illumina NovaSeq 6000 (San Diego, CA, USA, read 1: 28 bp and read 2: 91 bp). Library preparation and sequencing was performed by the Genomics Core, Cancer Research UK Cambridge Institute.

Single-cell clustering and differential gene expression analysis.

For the 10X generated NucSeq datasets, 10X Cellranger v6.0.1 was used to map sequence reads to the human genome GRCh38 and perform the UMI and gene-level counts against Ensembl gene model V100. The raw count matrices generated by the software were then used for downstream analyses. A downstream analysis on the raw count matrices was performed using the R v4.2.1 and Seurat v4.1.1[8]. Nuclei expressing less than 500 features, or less than 800 transcripts were removed as low-quality reads. Nuclei with more than 10000 different features were removed as these were likely doublets. Any nuclei expressing more than 5% mitochondrial RNA were excluded from the analysis. SCT normalization and variance stabilization of the data, using regularized negative binomial regression[9] in Seurat. The data was integrated prior to PCA, followed by unsupervised clustering analysis using the Louvain algorithm and Uniform Manifold Approximation and Projection (UMAP) dimension reduction. Marker genes for each cluster were identified using Wilcoxon's rank-sum test and receiver-operating curve (ROC) analyses. Pseudo-bulking and differential expression of genes between nuclei from the wild type and *BSN* p.L400W*fs*114* Het nuclei within each cluster was analysed using Negative Binomial GLM fitting and Wald statistics using the DESeq2 v1.3.6[10]. The p-values attained by the Wald test were corrected for multiple testing using the Benjamini and Hochberg method to generate adjusted p-values. Genes with a Log2FC of <-1 or >1 and with a p-value < 0.05 were selected for performing pathway analysis. The Metascape v3.5.20240101[11] pathway analysis was used to identify pathways

that were either upregulated or downregulated between the wild type and *BSN* p.L400W*fs*114* Het nuclei. Various downstream analyses were conducted using RStudio v2023.03.0+386, scDblFinder v1.11.4, tidyverse v1.3.2 and dplyr v1.0.9.

References

1. Pantazis, C. B. *et al.* A reference human induced pluripotent stem cell line for large-scale collaborative studies. *Cell Stem Cell* **29**, 1685-1702.e22 (2022).
2. Santos, D. P., Kiskinis, E., Eggan, K. & Merkle, F. T. Comprehensive Protocols for CRISPR/Cas9-based Gene Editing in Human Pluripotent Stem Cells. *Curr Protoc Stem Cell Biol* **38**, 5B.6.1-5B.6.60 (2016).
3. Skarnes, W. C., Pellegrino, E. & McDonough, J. A. Improving homology-directed repair efficiency in human stem cells. *Methods* **164–165**, 18–28 (2019).
4. Xue, Y. *et al.* GenEditID: an open-access platform for the high-throughput identification of CRISPR edited cell clones. *bioRxiv* 657650 (2019) doi:10.1101/657650.
5. Merkle, F. T. *et al.* Generation of neuropeptidergic hypothalamic neurons from human pluripotent stem cells. *Development* **142**, 633–643 (2015).
6. Kirwan, P., Jura, M. & Merkle, F. T. Generation and Characterization of Functional Human Hypothalamic Neurons. *Curr Protoc Neurosci* **81**, 3.33.1-3.33.24 (2017).
7. Dowsett, G. K. C. *et al.* A survey of the mouse hindbrain in the fed and fasted states using single-nucleus RNA sequencing. *Mol Metab* **53**, 101240 (2021).
8. Butler, A., Hoffman, P., Smibert, P., Papalexi, E. & Satija, R. Integrating single-cell transcriptomic data across different conditions, technologies, and species. *Nature Biotechnology 2018 36:5* **36**, 411–420 (2018).
9. Hafemeister, C. & Satija, R. Normalization and variance stabilization of single-cell RNA-seq data using regularized negative binomial regression. *Genome Biol* **20**, 1–15 (2019).
10. Love, M. I., Huber, W. & Anders, S. Moderated estimation of fold change and dispersion for RNA-seq data with DESeq2. *Genome Biol* **15**, 1–21 (2014).
11. Zhou, Y. *et al.* Metascape provides a biologist-oriented resource for the analysis of systems-level datasets. *Nature Communications 2019 10:1* **10**, 1–10 (2019).

Supplementary Figures

Supplementary Figure 1 | Exome-wide rare (MAF <0.1%) variant associations with BMI. (a) Combined Manhattan plot showing gene burden test results from BOLT-LMM algorithm using a linear mixed model (LMM, two-sided). The significance threshold is *P*<1.33x10-6 (0.05/37,691) after Bonferroni correction. Genes passing exome-wide significance (*P*<1.33×10^{-6}) are highlighted. Points are annotated with variant predicted functional class (MISS REVEL; missense variants with REVEL scores (above 0.5 or 0.7), HC PTV; high confidence protein truncating variants). (b) Combined QQ plot for the gene burden tests from BOLT-LMM algorithm using a linear mixed model.

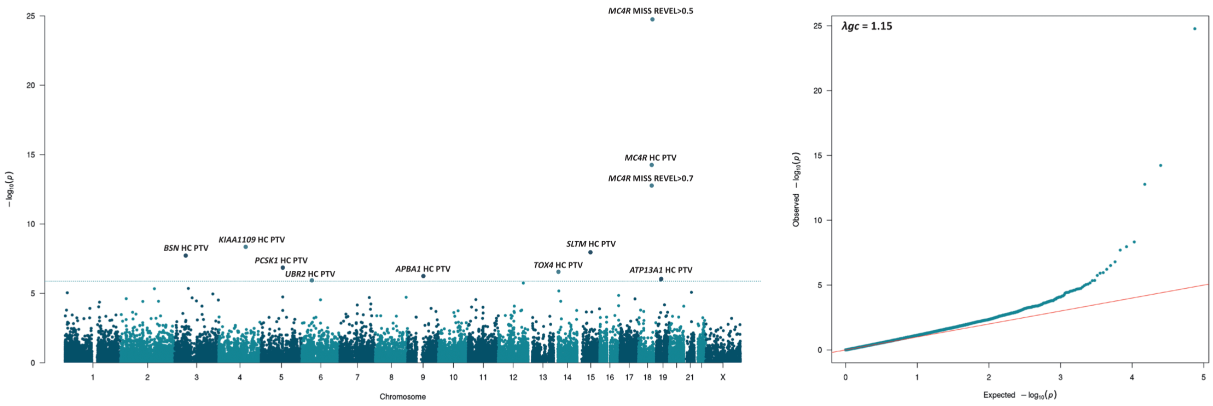

Supplementary Figure 2 | Exome-wide gene burden associations with BMI. QQ plots depict gene burden associations from BOLT-LMM algorithm using a linear mixed model for the three discovery variant masks and the synonymous variant mask as a negative control.

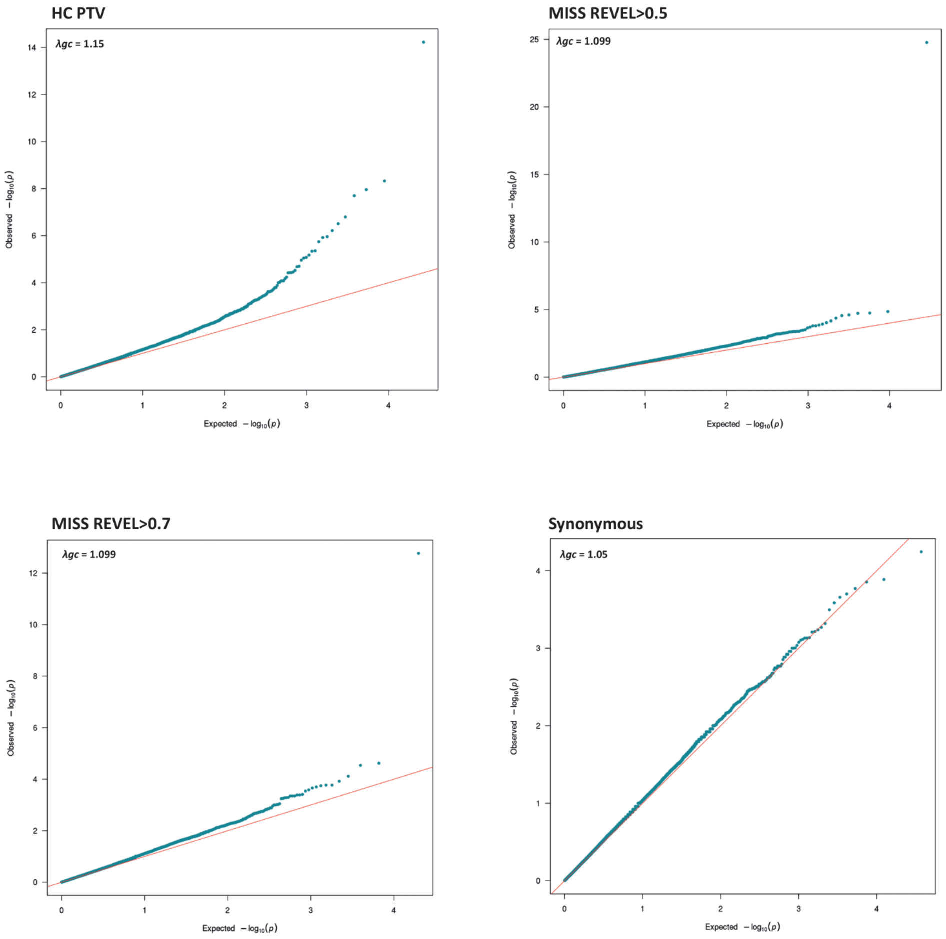

Supplementary Figure 3 | GWAS loci proximal to the exome-wide significant genes. Associations in the UK Biobank BMI GWAS from BOLT-LMM algorithm using a linear mixed model surrounding (±500kb) the 2 genes identified by exome-wide association with BMI, *BSN* (a) and *ATP13A1* (b). Relevant data are included in Table S2.

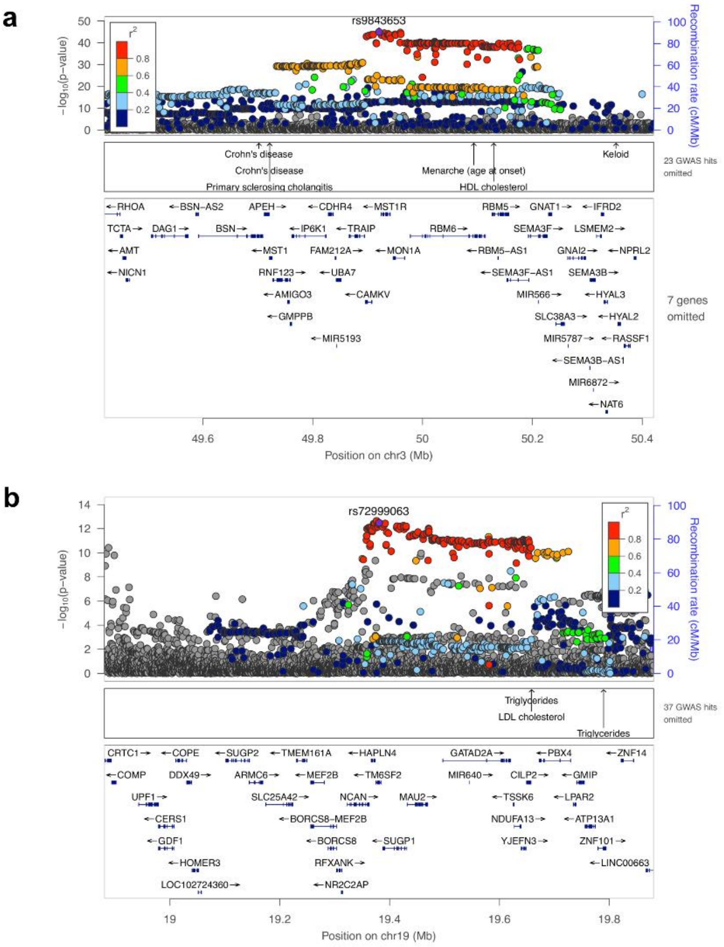

Supplementary Figure 4 | Replication of GWAS signals in Locke et al.[1] Associations from the UK Biobank BMI GWAS at the *BSN* (a) and *ATP13A1* (b) loci, were further queried in an independent GWAS cohort. Relevant data are included in Table S2.

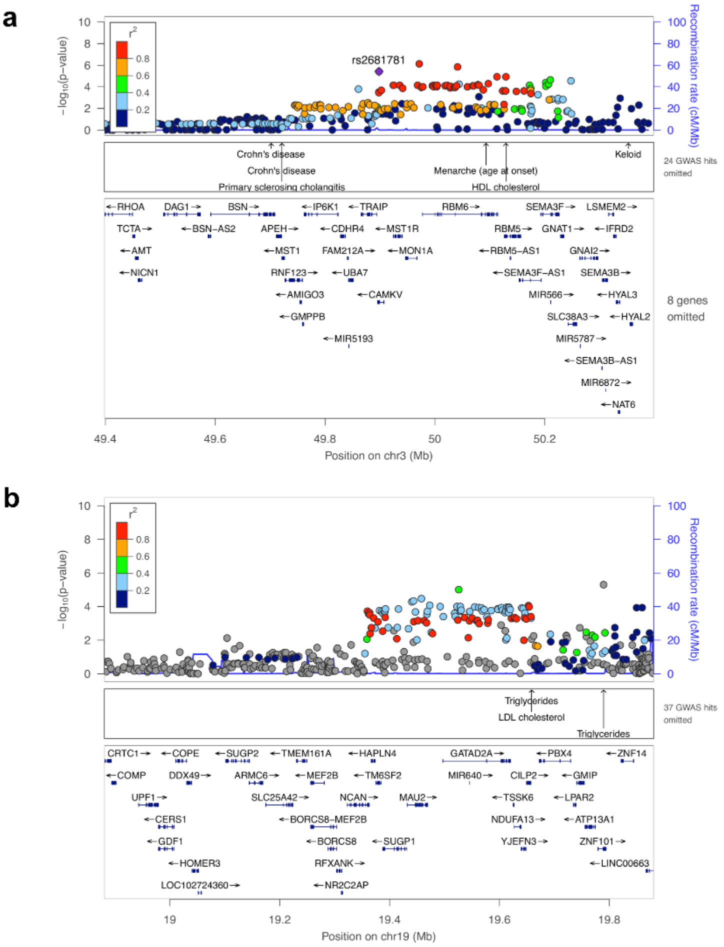

Supplementary Figure 5 | Geographical distribution of *APBA1* and *BSN* HC PTV carriers.

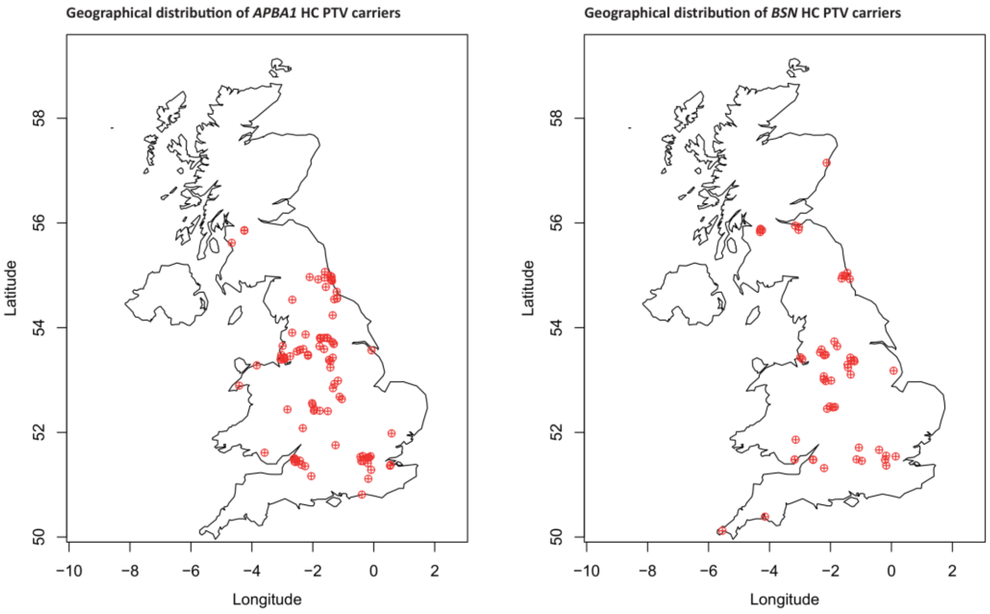

Supplementary Figure 6 | Interaction effect between PGS and *BSN* HC PTV carrier status. The effect of one standard deviation increase in PGS on BMI was approximately double in the carriers compared to non-carriers (0.61 vs. 0.30 in standard deviations), based on an interaction effect estimate of 0.31 (95% CI: 0.06-0.55, p=0.013, two-sided t-test). This was obtained from a linear regression model that included sex, age, age squared, genetic PC1 to PC10, PGS, carrier status, and the product of PGS and carrier status, with non-carrier as the reference group. The plot shows the predicted BMI in standard deviations for different PGS values in carriers and non-carriers, using the range of PGS values from the original data for predictions. The error band depicts the 95% confidence interval around the fitted BMI. HC PTV = high confidence protein truncating variant; PGS=polygenic score; SD=standard deviation.

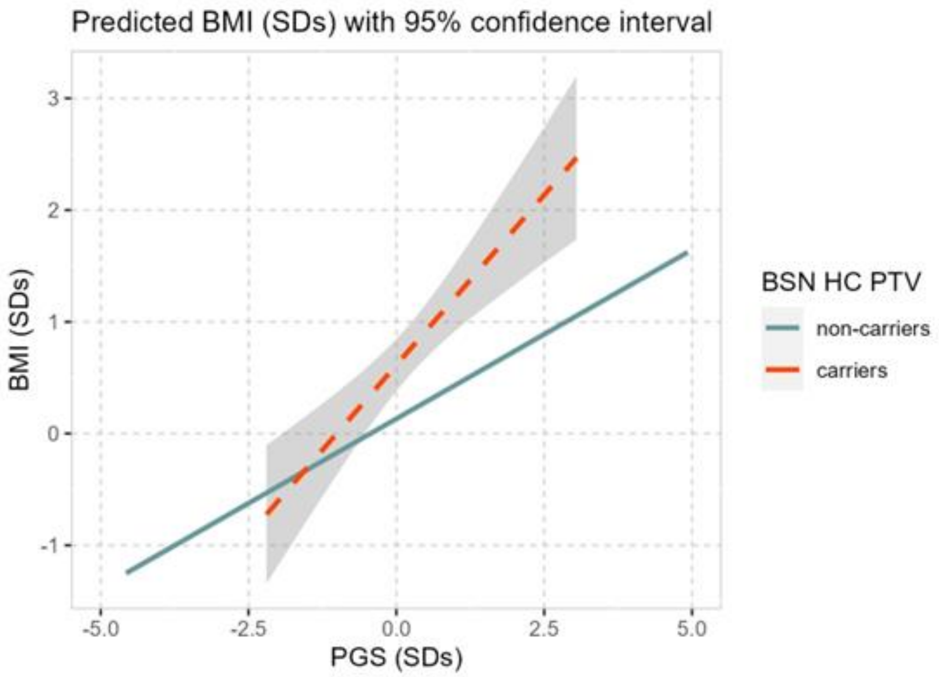

Supplementary Figure 7 | Kolf2 iPSCs differentiation into hypothalamic neurons. Kolf2 iPSCs are shown here on day 36 of differentiation. This differentiation has been repeated 3 times with the same results.

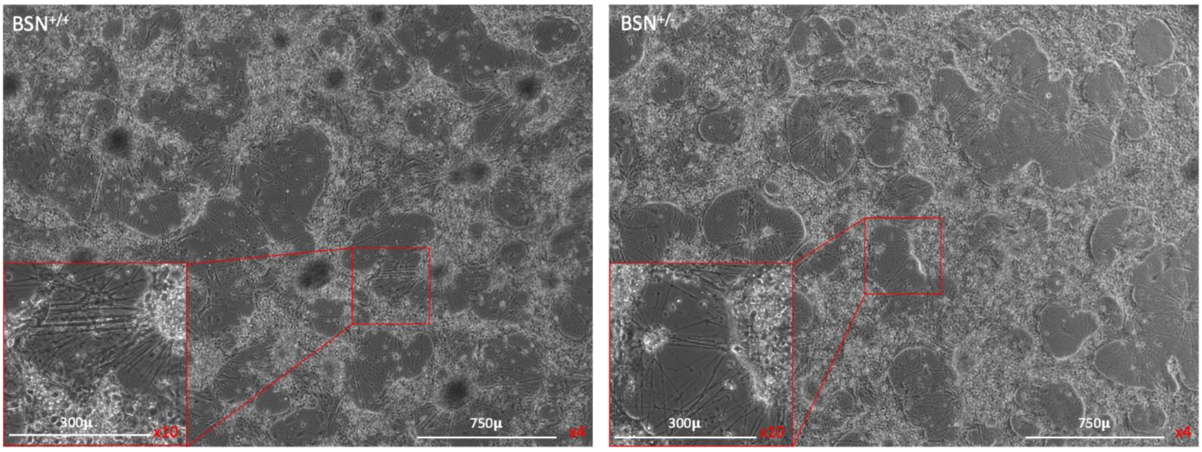

Supplementary Figure 8 | UMAP plots showing normalized expression of *RBFOX3*, *BSN, PCLO SLC1A3, GAD1 and SLC17A6*.

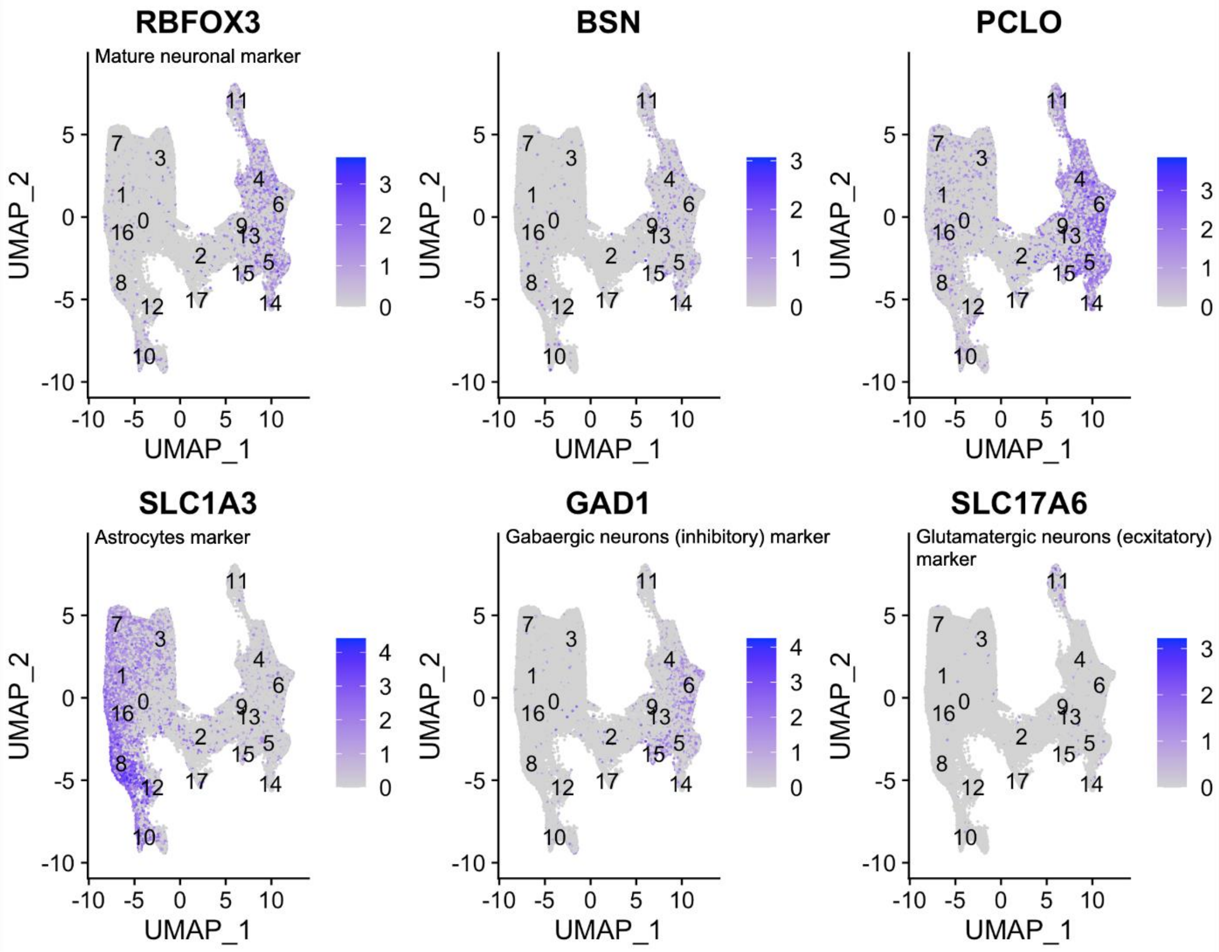

References

1. Locke, A. E. *et al.* Genetic studies of body mass index yield new insights for obesity biology. *Nature 2015 518:7538* **518**, 197–206 (2015).
