# Supplementary material for: Protein-truncating variants in BSN are associated with severe adult-onset obesity, type 2 diabetes and fatty liver disease
Source: Nat Genet. 2024 Apr 4;56(4):579–84. doi: 10.1038/s41588-024-01694-x (PMC11018524; doi:10.1038/s41588-024-01694-x)
Supplement: Supplementary file 2 — Reporting Summary [file 41588_2024_1694_MOESM2_ESM.pdf]

nature portfolio

Corresponding author(s): John R.B. Perry

Last updated by author(s): Jan 29, 2024

Reporting Summary

Nature Portfolio wishes to improve the reproducibility of the work that we publish. This form provides structure for consistency and transparency in reporting. For further information on Nature Portfolio policies, see our Editorial Policies and the Editorial Policy Checklist.

Statistics

For all statistical analyses, confirm that the following items are present in the figure legend, table legend, main text, or Methods section.

n/a	Confirmed	
☐	☒	The exact sample size (*n*) for each experimental group/condition, given as a discrete number and unit of measurement
☒	☐	A statement on whether measurements were taken from distinct samples or whether the same sample was measured repeatedly
☐	☒	The statistical test(s) used AND whether they are one- or two-sided<br>*Only common tests should be described solely by name; describe more complex techniques in the Methods section.*
☐	☒	A description of all covariates tested
☐	☒	A description of any assumptions or corrections, such as tests of normality and adjustment for multiple comparisons
☐	☒	A full description of the statistical parameters including central tendency (e.g. means) or other basic estimates (e.g. regression coefficient) AND variation (e.g. standard deviation) or associated estimates of uncertainty (e.g. confidence intervals)
☐	☒	For null hypothesis testing, the test statistic (e.g. *F*, *t*, *r*) with confidence intervals, effect sizes, degrees of freedom and *P* value noted<br>*Give P values as exact values whenever suitable.*
☒	☐	For Bayesian analysis, information on the choice of priors and Markov chain Monte Carlo settings
☒	☐	For hierarchical and complex designs, identification of the appropriate level for tests and full reporting of outcomes
☒	☐	Estimates of effect sizes (e.g. Cohen's *d*, Pearson's *r*), indicating how they were calculated

Our web collection on statistics for biologists contains articles on many of the points above.

Software and code

Policy information about availability of computer code

Data collection	GenEditID
Data analysis	Software: bcftools v1.14, R (v3.6.0, v3.6.3, v4.0.2, v4.2.1), ENSEMBL Variant Effect Predictor (VEP) (v96 (GRCh37), v104, v110), BOLT-LMM v2.3.6, bcl2fastq v2.19.0, Illumina DRAGEN Bio-IT Platform Germline Pipeline v3.0.7, SnpEff v4.3, KING v2.2.3, PEDDY v0.4.2, metafor v3.8-1, coloc v5.1.0, lassosum v4.0.5, Cellranger v6.0, 10X Cellranger v6.0.1, Seurat v4.1.1, DESeq2 v1.3.6, Metascape v3.5.20240101, RStudio v2023.03.0+386, scDblFinder v1.11.4, tidyverse v1.3.2, dplyr v1.0.9<br><br>Algorithm: regularized negative binomial regression, Louvain algorithm, Uniform Manifold Approximation and Projection (UMAP) dimension reduction, Wilcoxon's rank-sum test, receiver-operating curve (ROC) analyses, Negative Binomial GLM fitting, Wald statistics, Benjamini and Hochberg method

For manuscripts utilizing custom algorithms or software that are central to the research but not yet described in published literature, software must be made available to editors and reviewers. We strongly encourage code deposition in a community repository (e.g. GitHub). See the Nature Portfolio guidelines for submitting code & software for further information.

Data

Policy information about availability of data

All manuscripts must include a data availability statement. This statement should provide the following information, where applicable:

- Accession codes, unique identifiers, or web links for publicly available datasets
- A description of any restrictions on data availability
- For clinical datasets or third party data, please ensure that the statement adheres to our policy

The UK Biobank phenotype and whole-exome sequencing data described here are publicly available to registered researchers through the UK Biobank data access protocol. Information about registration for access to the data is available at: https://www.ukbiobank.ac.uk/enable-your-research/apply-for-access. Data for this study were obtained under Resource Applications 26041 and 9905. The Mexico City Prospective Study welcomes open access and collaboration data requests from bona fide researchers. For more details on accessibility, the study's Data and Sample Sharing policy may be downloaded (in English or Spanish) from https://www.ctsu.ox.ac.uk/research/mcps. Available study data can be examined in detail through the study's Data Showcase, available at https://datashare.ndph.ox.ac.uk/mexico/. SCOOP and INTERVAL whole-exome sequencing data are accessible from the European Genome-phenome Archive with accession numbers EGA: EGAS00001000124 (SCOOP) and EGA: EGAS00001000825 (INTERVAL). The single-nucleus RNA sequencing data is available from the NCBI Gene Expression Omnibus (GEO), accession number: GSE243112.

Research involving human participants, their data, or biological material

Policy information about studies with human participants or human data. See also policy information about sex, gender (identity/presentation), and sexual orientation and race, ethnicity and racism.

Reporting on sex and gender	In our analyses, we included both males and females and we adjusted sex in our regression analysis.
Reporting on race, ethnicity, or other socially relevant groupings	In UK Biobank, we restricted our analysis to European ancestry, and we defined a subset of European ancestry samples using a k-means-clustering approach that was applied to the first four principal components calculated from genome-wide SNP genotypes.
Population characteristics	The UK Biobank is a large prospective cohort that recruited approximately 500,000 participants aged 40 to 69 years across the island of Great Britain. A broad range of phenotypic and health-related information was collected from each participant, including physical measurements, lifestyle indicators, biomarkers in blood and urine, imaging, and routine health record data.<br><br>The Mexico City Prospective Study is a cohort study of 159,755 adults (mean age 52.6years and 67.26% are females) of predominantly Admixed American ancestry. Phenotypic data were recorded during household visits, including height, weight, and waist and hip circumferences. Disease history was self-reported at baseline, and participants are linked to Mexican national mortality records.<br><br>The Pakistan Genomic Resource study has been recruiting participants aged 15-100 years (mean age 54.25 years and 34.95% are females) as cases or controls via clinical audits for specific conditions since 2005 from over 40 centres around Pakistan. DNA, serum, plasma, and whole-blood samples were also collected from all study participants.<br><br>The Genetics of Obesity Study (GOOS) (SCOOP cohort) contains 927 White British participants with severe early-onset obesity. All participants had age < 10y at the time of recruitment, sex distribution was: Female 548 (59.12%), Male 379 (40.88%).<br><br>INTERVAL cohort contains 4,057 UK blood donors. Information on age and sex was available to us for 4,045 of the 4,057 participants (99.70%): Age mean (SD): 43.51 (14.31); Sex Female 1,994 (49.30%), Male 2,051 (50.70%).
Recruitment	Participants of the UK Biobank aged from 40 to 69, who were registered with NHS and living up to about 25 miles from one of the 22 study assessment centres were invited to participate in 2006-2010.<br><br>Participants of the MCPS study were recruited between 1998 and 2004 aged 35 years or older from two adjacent urban districts of Mexico City.<br><br>Participants of the Pakistan Genomic Resource study were recruited from clinics treating patients with cardiometabolic, inflammatory, respiratory, or ophthalmological conditions. Information on lifestyle habits, medical and medication history, family history of diseases, exposure to smoking and tobacco consumption, physical activity, dietary habits, anthropometry, basic blood biochemistry and ECG traits were recorded during clinic visits.<br><br>SCOOP comprises UK patients with severe obesity (BMI>+3 SD for age and sex) of early onset (<10 years) recruited to the Genetics of Obesity Study (GOOS).<br><br>INTERVAL comprises predominantly healthy blood donors in the UK (https://www.intervalstudy.org.uk).
Ethics oversight	The UK Biobank has approval from the North West Multi-centre Research Ethics Committee (REC reference 13/NW/0157, https://www.ukbiobank.ac.uk/media/lcvbdoik/21-nw-0157-favourable-opinion-with-conditions-18-06-2021.pdf) as a Research Tissue Bank (RTB) approval and informed consent (https://www.ukbiobank.ac.uk/media/t22hbo35/consent-

form.pdf) was provided by each participant. This approval means that researchers do not require separate ethical clearance and can operate under the RTB approval. This RTB approval was granted initially in 2011 and it is a renewal on a 5-yearly cycle; hence UK Biobank successfully applied to renew it in 2016 and 2021.

The MCPS study was approved by the Mexican Ministry of Health, the Mexican National Council for Science and Technology, and the University of Oxford.

The Pakistan Genomic Resource study was approved by the institutional review board at the Center for Non-Communicable Diseases (IRB: 00007048, IORG0005843, FWAS00014490) the study and all participants gave informed consent.

SCOOP were approved by the Multi-Regional Ethics Committee and the Cambridge Local Research Ethics Committee (MREC 97/21 and REC number 03/103). Participants (or parents for those <16 years) provided written informed consent; minors provided oral consent. INTERVAL study was approved by National Research Ethics Service approved (11/EE/0538), whose participants provided informed consent before joining the study.

Note that full information on the approval of the study protocol must also be provided in the manuscript.

Field-specific reporting

Please select the one below that is the best fit for your research. If you are not sure, read the appropriate sections before making your selection.

☒ Life sciences ☐ Behavioural & social sciences ☐ Ecological, evolutionary & environmental sciences

For a reference copy of the document with all sections, see nature.com/documents/nr-reporting-summary-flat.pdf

Life sciences study design

All studies must disclose on these points even when the disclosure is negative.

Sample size

We used the full available sample with whole-exome sequencing data in UK Biobank (N=454,787) for discovery analyses.

Both wild type cells and cells heterozygous for a BSN mutation (P399X; BOLT ID 3:49642828:D:1) were grown and differentiated in 6 well plates, without inter well cross-contamination. Each well was treated as a separate sample, hence N=3 for wild type cell samples and N=9 for heterozygous cell samples. Sequencing libraries for the 6 (3 wild type and 9 heterozygous) single-nuclei suspension samples were then generated separately using 10X Genomics Chromium Single-Cell 3'V3.1 Reagent kits (Pleasanton, CA, USA) according to the standardised protocol. The sample size was not pre-determined as there were no studies on the effects of BSN prior to this paper. We employed a 2-step approach and determined the N based on the variance observed in the data obtained from the initial experiment.

Data exclusions

Only individuals failing standard genotyping quality control parameters defined initially by the UK Biobank study, individuals of non-European ancestry or with missing phenotype or covariates were excluded from analysis. This decision was made prior to performing any downstream analysis.

Replication

We replicated findings in two independent studies (total N=178,846). All attempted replication has been reported in the manuscript without exception.

Randomization

The principle exposure in this study is a naturally occurring genetic variant, meaning that we were unable to randomize the individuals in the study. To account for possible confounding, we used a linear mixed model and adjusted for technical and demographic covariates.

Blinding

This study is not a randomized controlled trial. We didn't give any intervention to the participants in this study. Blinding is not applicable to this study.

Reporting for specific materials, systems and methods

We require information from authors about some types of materials, experimental systems and methods used in many studies. Here, indicate whether each material, system or method listed is relevant to your study. If you are not sure if a list item applies to your research, read the appropriate section before selecting a response.

Materials & experimental systems

n/a	Involved in the study	
☒	☐	Antibodies
☐	☒	Eukaryotic cell lines
☒	☐	Palaeontology and archaeology
☒	☐	Animals and other organisms
☒	☐	Clinical data
☒	☐	Dual use research of concern
☒	☐	Plants

Methods

n/a	Involved in the study	
☒	☐	ChIP-seq
☒	☐	Flow cytometry
☒	☐	MRI-based neuroimaging

Eukaryotic cell lines

Policy information about cell lines and Sex and Gender in Research

Cell line source(s)	Human Kolf2.1 J induced pluripotent stem cells were sourced in-house at the Institute of Metabolic Science, University of Cambridge, United Kingdom. There is no commercial source, we inherit the cell line from the Merkle Lab (fm436@medschl.cam.ac.uk).
Authentication	The cell lines were not authenticated.
Mycoplasma contamination	All cell cultures were tested for the presence of Mycoplasma prior to use, and subsequently tested at regular intervals during the experiments. No mycoplasma was detected by any of the tests. Testing was performed using the EZ-PCR Mycoplasma Kit (BI Biological Industries, 20-700-20), according to the manufacturer's instructions.
Commonly misidentified lines (See ICLAC register)	This is not a commonly misidentified cell line.
